# Using dried blood spot samples from a trio for linked-read whole-exome sequencing

**DOI:** 10.1038/s41431-019-0343-3

**Published:** 2019-02-14

**Authors:** Ólavur Mortensen, Leivur Nattestad Lydersen, Katrin Didriksen Apol, Guðrið Andorsdóttir, Bjarni á Steig, Noomi Oddmarsdóttir Gregersen

**Affiliations:** 1 FarGen, The Genetic Biobank of the Faroe Islands, Tórshavn, Faroe Islands; 2 General Medical Department, National Hospital of the Faroe Islands, Tórshavn, Faroe Islands

**Keywords:** Genetics research, Haplotypes

## Abstract

Long-term collection of dried blood spot (DBS) samples through newborn screening may have retrospective and prospective advantages, especially in combination with advanced analytical techniques. This work concerns whether linked-reads may overcome some of the limitations of short-read sequencing of DBS samples, such as performing molecular phasing. We performed whole-exome sequencing of DNA extracted from DBS and corresponding whole blood (WB) reference samples, belonging to a trio with unaffected parents and a proband affected by primary carnitine deficiency (PCD). For the DBS samples we were able to phase >21% of the genes under 100 kb, >40% of the SNPs, and the longest phase block was >72 kb. Corresponding results for the WB reference samples was >85%, >75%, and >915 kb, respectively. Concerning the PCD causing variant (rs72552725:A > G) in the *SLC22A5* gene we observe full genotype concordance between DBS and WB for all three samples. Furthermore, we were able to phase all variants within the *SLC22A5* gene in the proband’s WB data, which shows that linked-read sequencing may replace the trio information for haplotype detection. However, due to smaller molecular lengths in the DBS data only small phase blocks were observed in the proband’s DBS sample. Therefore, further optimisation of the DBS workflow is needed in order to explore the full potential of DBS samples as a test bed for molecular phasing.

## Introduction

Whole-exome sequencing (WES) is increasingly being used in the clinic for diagnostic evaluation and variant detection, particularly for genetically heterogeneous diseases [1, 2]. The use of trio information in this context has further improved diagnostic yield as haplotype information is obtained and novel candidate genes can be detected on the basis of inheritance consistency [3]. The ability to obtain haplotype resolved sequences has aided the genetic interpretation of WES data (e.g., compound heterozygosity), and is also critical for providing insight into complex disease aetiologies [4]. This has inspired the development of assays for molecular phasing [5–7], including linked-read sequencing, which has further improved the alignment of short-reads by introducing long-range information.

Together with the emergence of advanced analytical techniques the use of dried blood spot (DBS) samples as a primary testing source has also broadly expanded. Researchers worldwide are exploring the retrospective and prospective advantages of the long-term collection of DBS samples through newborn screening (NBS) programs in combination with next-generation sequencing (NGS) [8–11]. The National Hospital of the Faroe Islands has collected DBS samples since 1986; the current 22,000 DBS cards may be a valuable source for the recent precision medicine initiative of the Faroese Biobank, which may give valuable insight into heritable traits when whole-blood (WB) samples are otherwise unavailable. Primary carnitine deficiency (PCD), with a prevalence of 1:300 [12] in the Faroese isolate, is one of the traits included in the NBS program of the Faroese health care system. This recessive autosomal disorder is caused by homozygous or compound heterozygous variants in the *SLC22A5* gene. Genetic studies of Faroese individuals show four different causative founder variants within this population, where homozygotes for the rs72552725:A > G variant have the most severe phenotype [13].

Using DBS samples for NGS analyses can be challenging due to degradation and the limited quantities of DNA obtained from each card, which may be inadequate for some NGS platforms. However, it has previously been shown that DNA extracted from DBS cards without whole-genome amplification can be used for WES studies [14], and that causal variants for inherited metabolic diseases can be detected [11]. As short reads have their own set of limitations, we investigate the possibility of using DBS samples together with linked-read sequencing for detection of the disease-causing variant rs72552725 in PCD, and whether this approach may replace the trio information for haplotype detection. Here we perform WES of a trio—two unaffected parents and one child affected with PCD using DBS and corresponding WB reference samples in order to investigate the feasibility of performing molecular phasing with archival samples.

## Materials and methods

### Individuals, blood/DBS sampling and DNA extraction

The trio was recruited to the FarGen infrastructure (www.fargen.fo), at the Genetic Biobank of the Faroe Islands and the child’s self-reported phenotype was confirmed by the diagnostic registry at the National Hospital of the Faroe Islands. All three individuals have given written consent to genetic research, which has been approved by the research ethics committee of the Faroe Islands. Peripheral blood samples were collected in EDTA tubes and stored as WB at −40 °C until DNA extraction. In order to obtain a DBS sample from the trio the collected EDTA-treated blood (50 µL) was spotted onto Whatman FTA cards (Whatman INC., Brentford, UK) and dried for 48 h at room temperature until DNA extraction.

DNA was extracted from three DBS punches (6 mm in diameter) using the Chemagic Prepito-D according to manufacturer’s protocol (PerkinElmer, Waltham, MA, USA), with minor alterations—the tubes were mixed for 5 s and then incubated at room temperature for 2 h with agitation. Further, DNA was extracted from 250 µL WB using the Chemagic Prepito-D (PerkinElmer). All DNA extractions were performed at the laboratory of the National Hospital, Tórshavn, Faroe Islands.

### DNA barcoding and library construction

DNA concentrations were normalized with Low TE buffer to the recommended concentration between 0.8–1.2 ng/µl. Libraries were prepared using the Chromium^TM^ Genome Chip Kit v2 following the manufacturer’s exome demonstrated protocol (10xGenomics, Pleasanton, CA, USA) and barcoded using the Chromium Controller Instrument (10xGenomics). By partitioning the DNA samples into >1,000,000 droplets in oil emulsion (GEMs), the preparations of sequencing libraries happen in parallel, introducing 16-bp specific barcodes onto the DNA fragments. Library yield and fragment sizes were determined using the Agilent 2100 Bioanalyzer (Agilent, Santa Clara, CA, USA). Samples were sheared to an average size of 300 bp using the Covaris M220 system (Covaris, Woburn, MA, USA). Further preparation of the libraries was conducted in accordance with the 10xGenomics manual (10xGenomics).

### Whole-exome capturing and sequencing

Exomes were captured using the SureSelectXT Human All Exon v6 kit (Agilent) following the 10xGenomics protocol (10xGeneomics, Pleasanton, CA, USA). A total of six exomes were sequenced on the Illumina NextSeq 500 platform (Illumina^®^, San Diego, CA, USA) at the Research Park iNOVA, Tórshavn, Faroe Islands.

### Alignment

Linked-reads were aligned to the hg19/GRCh37 reference genome using the LongRanger v2.1.5 pipeline [7]. The reads were aligned to target regions (SureSelect Human All Exon v6) with a 100 bp padding on each side of the exons. The first 10 bp of the reads were trimmed prior to alignment. The linked-reads were aligned using the Lariat aligner [7], which uses BWA version 0.7.12 [15] to generate alignment candidates, and duplicate reads are marked after alignment [16].

### Variant calling and haplotype phasing

SNPs and indels were called from the aligned BAM files with the LongRanger pipeline, which calls FreeBayes version 0.9.21 [17] applying default parameters. Each sample was called separately. After variant calling, LongRanger annotated the variants with barcodes and phased the haplotypes of the heterozygous genotypes.

### Functional annotation

All variants’ functions are determined with SnpEff (v4.3r) [18]. Variants were classified e.g., as missense, synonymous, nonsense, frameshift, various splice site variants, various UTR variants. Based on the functional annotation, the variants are classified in terms of impact as high (loss of function), moderate (possible change in effectiveness), low (unlikely to change protein behaviour) or modifier (no evidence of impact).

### Mendelian inheritance errors (MIEs)

MIEs are errors in variants that arise when the child cannot have inherited one or both of the alleles from his/her parents; e.g., if both parents are homozygous with the reference, and the child is heterozygous, then there is an MIE. We calculate MIEs in the trio using PLINK [16]. All types of MIE are considered, including when one sample has missing genotypes.

### Concordance

Two VCFs are considered concordant for a variant if the genotypes are identical. In our analyses, only overlapping variants are considered, so variants that are present in only one of the VCFs are not included. The concordance rate is the ratio of the number of concordant variants to the total number of overlapping variants $concordance\;rate = \frac{{matches}}{{matches + mismatches}}$.

## Results

### Individuals and DNA extraction

The collected peripheral blood samples from the trio were used to extract DNA from DBS, which had been dried for 48 h at room temperature, and corresponding WB reference samples; the three DBS punches from father, mother and proband yielded 88 ng, 148 ng and 444 ng DNA, respectively. Corresponding WB reference samples yielded 1042, 1320 and 4129 ng DNA, respectively. Before library preparation DNA was barcoded by the gel-bead emulsion (GEM) process in the Chromium^™^ controller. Due to the barcodes introduced to the DNA fragments during GEM formation, we are able to obtain molecular length measurements (Table 1). Average molecule lengths of the DNA extracted from WB was ~ 50 kb, which is in the recommended range for linked-read sequencing, with between 80–84% of the DNA in molecules >20 kb, and between 7–13% in molecules >100 kb. Corresponding results for the DBS samples was ~10 kb, which is somewhat below the recommended range, with between 5 and 14% of the DNA in molecules >20 kb and 0.42–1.29% in molecules >100 kb. Estimated DNA loaded was within the recommended range (0.8–1.2 ng/µl) for the WB samples, while for the DBS samples < 0.30 ng DNA was estimated. However, these loading estimates, and other metrics calculated by the LongRanger pipeline [7] (Table 1) are tuned for samples with molecule length within the recommended range and may incorrectly estimate length when outside this range.

**Table 1:** Results from linked-read whole-exome sequencing of a trio using dried blood spots and whole-blood samples

	Whole blood				Dried blood spots			
	Father	Mother	Proband	Average	Father	Mother	Proband	Average
*Inp*ut *DNA*								
Median molecule length (bp)	45,239	47,415	55,129	49,261	11,155	2425	15,643	9741
% DNA in molecules >20 kb	80	82	84	82	14	5	12	11
% DNA in molecules >100 kb	7	13	13	11	0.42	1.29	0.75	0.82
DNA loaded (ng)	1.14	1.58	1.17	1.30	0.20	0.29	0.30	0.26
GEM Performance								
GEMs detected	1,472,690	1,627,223	1,450,403	1,516,772	1,236,375	1,235,548	1,124,027	1,198,650
Mean DNA per GEM (bp)	488,116	673,692	501,609	554,472	86,500	122,798	128,697	112,665
*Sequencing and alignment*								
Number of reads	102,043,815	146,325,136	87,244,491	111,871,147	134,915,555	111,553,510	110,707,511	119,058,859
Fold coverage	66	94	56	72	77	67	61	68
Zero coverage	0.19	0.37	0.62	0.39	0.85	1.23	1.24	1.11
Median insert size (bp)	197	204	183	195	166	172	184	174
% bases on target	60	58	59	59	60	60	55	58
% fragments on target	60	59	59	59	60	60	56	59
% mapped reads	98	98	98	98	94	96	96	95
Avg. mapping quality	59	56	56	57	52	53	54	53
% duplication	17	24	28	23	40	43	45	43

### Whole-exome sequencing of the trio using DBS and WB samples

Overall sequencing performance for the DBS and WB samples were uniform for most parameters, though some variance is seen between the samples (Table 1). The six exomes were sequenced with an average coverage depth of between 28x and 72x, >94% of the reads were aligned to the reference genome (GRGh37/hg19), >55% of the bases were on target, and median insert size between 166 and 204 bp. Mean duplication rate was 43% for DBS and 23% for WB, which likely is due to less DNA was loaded from the DBS samples.

### Variant detection and phasing

**Fig. 1:**
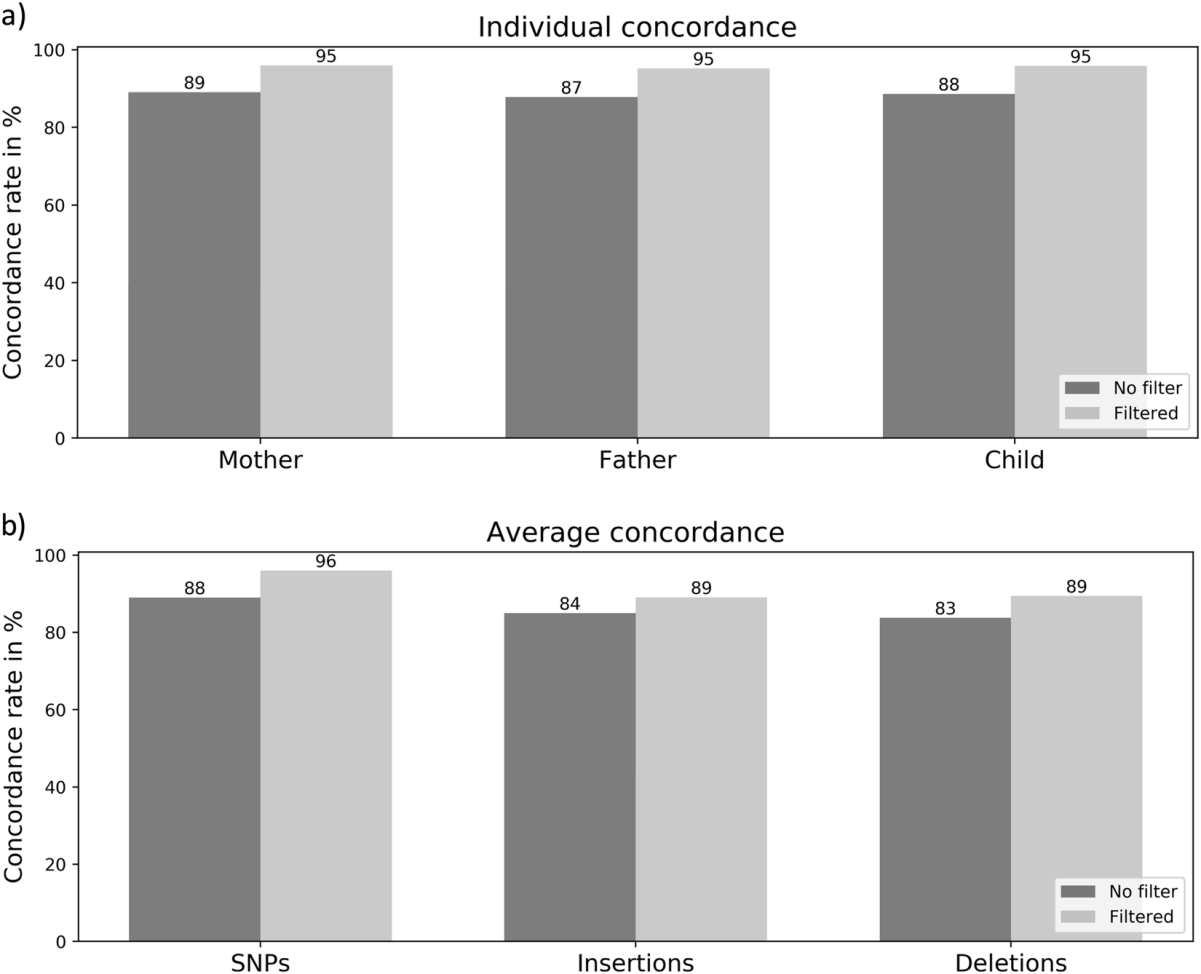
Genotype concordance rate between whole-blood (WB) and corresponding dried blood spot (DBS) samples is shown individually (**a**) and averaged overall three samples for SNPs, insertions, and deletions (**b**). Filtered variants are filtered according to genotype quality >20 and read depth >20, and multi-allelic variants are removed

Table 2 gives the number of variants called from the DBS and WB data sets using the LongRanger pipeline [7]. The total number of variants is the union of all variants seen in at least one member of the trio. After filtering variants to include only those with read depth >20, variant call quality >20, and eliminating multi-allelic sites the total number of variants was 219,586 in the WB samples and 245,736 in the DBS samples. The number of variants overlapping between WB and DBS was 209,931, meaning that 96% of the variants in the WB data are also present in the DBS data. Pairwise comparison of genotypes (SNPs, insertions and deletions) in the WB and DBS data sets showed a concordance rate of 96% for SNPs and 89% for indels (Fig. 1), which is in range with previously reported rates [19]. The trio information allows us to identify Mendelian inheritance errors (MIEs) i.e., variants in the proband that could not have been inherited from the parents. Filtered variants with MIE in the proband was 0.38% in the WB data and 1.2% in the DBS data, which reflects the slightly lower quality of the DBS data set.

**Table 2:** Number of variants called from whole-blood (WB) and dried blood spots (DBS) samples from the trio

	WB				DBS				
	Father	Mother	Proband	Total	Father	Mother	Proband	Total	Overlap
Variants called	592,661	747,825	591,139	1,538,193	898,132	726,946	862,282	2,135,974	430,865
Filtered variants*	161,705	171,137	162,323	219,586	163,433	160,447	162,179	245,736	209,931
Variants by impact									
High	842	928	896	1219	1361	1415	1386	2804	1146
Moderate	11.254	11,683	11,328	15,583	12,897	12,853	12,368	21,149	14,855
Low	14,988	15,616	15,186	20,413	15,212	15,175	14,883	22,731	19,627
Modifier	134,621	142,910	134,913	182,371	133,963	131,004	133,542	199,052	174,303
*Phasing*									
SNPs phased (%)	81	84	75		58	40	49		
Genes phased (<100 kb) (%)	92	94	85		42	21	33		
Longest phase block (kb)	938	2 071	1 058		149	116	100		
N50 phase block (kb)	124	176	113		13	4	11		

The total number of variants corresponds to variants seen in at least one of the individuals in the trio. The overlap is the number of variants present in the total DBS and WB data sets. Phasing summary is based on filtered variants only

*Filters: read depth >20, variant call quality >20, and multi-allelic sites removed

Variants were annotated using SnpEff [18] and are here reported by impact, where *High* represents variants predicted to have a deleterious effect on protein structure (e.g., nonsense and frameshift variants) (see Materials and methods section). The total number of high impact variants was 1219 and 2804 for WB and DBS, respectively, and 1146 of these variants were present in both data sets, in other words, 94% of the high impact variants present in the WB data were also present in the DBS data. However, due to the higher number of variants called in the DBS data this overlap is reduced to 41% when comparing DBS variants also present in the WB data. The low overlap might suggest lower quality of the DBS samples; and further analysis confirmed this when applying a more stringent filter (read depth >60 and variant call quality >60) 37% of the high impact variants in the DBS data were filtered out and only 16% in the WB data (data not shown).

In this study, we define a phase block as a region of the genome stretching from the leftmost phased heterozygote to the rightmost phased heterozygote in a phase set. For the WB exome data, the phasing algorithm was able to phase >85% of the genes under 100 kb, >75% of the called SNPs were phased, the longest phase block was >915 kb, and we observed N50 phase block lengths between 105 and 170 kb. For the DBS exome data >21% of the genes under 100 kb were phased, >40% of the SNPs were phased, the longest phase block was >72 kb, and N50 phase block lengths were between 2 kb and 11 kb (Table 2).

### The *SLC22A5* gene

**Fig. 2:**
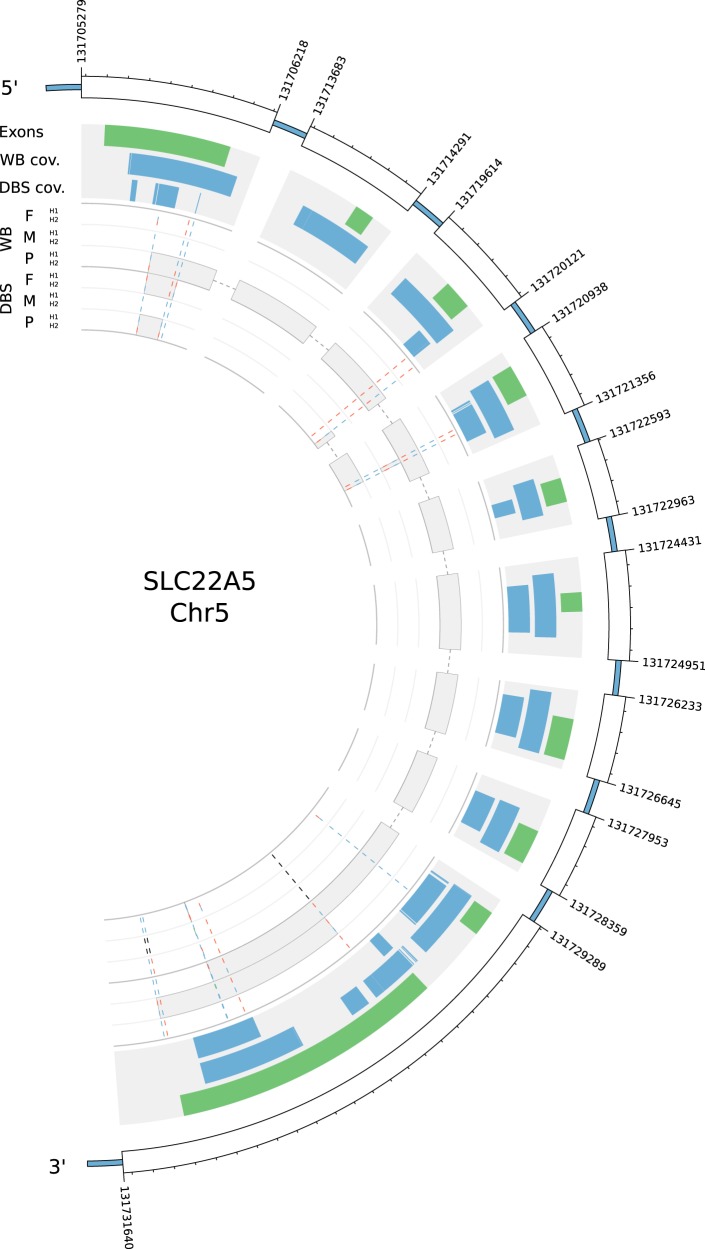
The plot shows coverage and variants in the *SLC22A5* gene using data from the WB and DBS trio samples. The row with green bands correspond to exons of transcript uc003kww.4 (UCSC identifier) of the *SLC22A5* gene. Regions in between the exons are excluded from the plot to increase clarity. The two rows with blue bands are coverage in the WB and DBS data sets. Only regions where all three samples have coverage >20x are shown. Variants (variant call quality >20 and read depth >20x ) within the *SLC22A5* gene identified in the trio (F = father, M = mother, P = proband) are shown in the inner rows (WB and DBS); alleles are colour coded as follows: reference allele = blue and alternate alleles = red or green (random order), no calls = black. Phase blocks are shown in the inner rows as grey boxes (note that phase blocks spanning multiple exons are connected with dotted lines). Heterozygous variants that are not phased are shown closer together in the middle of the band. H1 = haplotype 1, and H2 = haplotype 2. The plot was generated using Circos [20]

Due to the phenotype of the proband, we explored the *SLC22A*5 gene in more detail. Figure 2 illustrates the exons (green bands), coverage (blue bands) of the gene, and identified variants in the gene. Only regions where all three samples have a coverage >20 are shown in the WB and DBS data sets, respectively. Overall, we observe better coverage of the gene in the WB data, which is consistent with the higher overall coverage observed in the WB samples (Table 1). After filtering variants according to read depth >20 and variant call quality >20 (keeping multi-allelic variants) we observed 14 variants within *SLC22A*5 (Fig. 2 and supplementary Table 1). Eight of these variants were called as heterozygotes in both data sets of the proband, including rs72552725 (NG_008982.1:g.5359A > G), which further was the only variant classified as a high impact variant by SnpEff. Rs72552725 was the only of fourteen variants called as heterozygote in the fathers WB data, while in the DBS data four more heterozygote calls were observed. In the mothers WB data set five of fourteen variants were called as heterozygotes, while in the DBS data a single heterozygote call was observed; rs72552725 was called as homozygote with the reference in both data sets of the mother. We see some discordance between genotype calls in the WB and DBS data of the trio, further, several of the variants were not called (no call), and a single MIE was detected in the multi-allelic position rs144261584;rs14701 (NC_000005.9:g.131730923_131730924insC;NG_008982.1:g.30522A > C) (Supplementary Table 1).

The molecular phasing is performed using the barcodes introduced to the DNA during GEM formation; for convenience we will refer to molecular phasing as phasing only. Phase blocks of heterozygous variants called in *SLC22A5* are shown as grey boxes within the inner variant band in Fig. 2. Non-phased heterozygous variants are shown closer together in the middle of the band (see rs72552725 in the fathers WB data). In the proband’s WB data we observe a phase block spanning most of the target region of the *SLC22A5* gene, the two haplotypes H1 and H2 are based on the eight heterozygous variants called in the probands data, including rs72552725. Moreover, all alternate alleles are on the H2 haplotype, inherited from the father. Only smaller phase blocks were observed in the proband’s DBS data, which may be due to the smaller molecule lengths seen in this data. In the mother’s WB data, a single phase block spanning five heterozygous variants at the 3’end of the gene was observed; no phase block was observed in the mother’s DBS data mainly due the to lack of heterozygote calls. Moreover, no phase block was observed in the fathers WB data due to rs72552725 being the only heterozygous variant called. In the fathers DBS data, a single phase block between two heterozygote variants was observed, including rs72552725 and a variant called as homozygous in the WB data.

## Discussion

This study investigated the ability to perform molecular phasing with DBS samples and explored the possibility to use such samples together with linked-reads for detection of a disease-causing variant (rs72552725) in PCD. The study comprises whole-exomes of a PCD case-unaffected parent’s trio obtained from DBS and corresponding WB reference samples. From a bioinformatics perspective, the benefit of using linked-reads is that we gain long-range information from short read sequencing, which could give a more accurate assembly and the ability to correctly assign the haplotypes of the variants; advantages that may especially apply to DBS samples as these samples may be more degraded than WB samples.

One of the challenges working with DBS samples is to obtain sufficient amount of DNA required for NGS analyses, as well as obtaining high molecular weight DNA as DBS samples may be degraded. In this study, we were able to obtain the recommended 1 ng of DNA from the DBS to perform the Chromium barcoding and WES analysis. Even though we were aiming for the recommended amount of DNA, the results showed that an inadequate amount of DNA was loaded for the DBS samples. This may be due to algorithmic limitations as the Chromium protocol is optimised for WB samples, and therefore the estimated DNA loaded can be inaccurate detected when short molecules are used as seen in the DBS samples (Table 1), the average molecular lengths of the DNA in the DBS samples are somewhat below the recommended range. This may have influenced the overall performance of the DBS samples by lower coverage and high duplication rates, which suggests that optimisations are required for the workflow of DBS samples in order to obtain better yield and better quality of data, especially when using older archive samples. This should include the automated process in which the DNA is extracted e.g., by adjusting the machine to handling the DBS samples even more gentle in order to keep the already degraded DNA molecules intact, optimisation of the Chromium protocol for DBS samples e.g. loading more than the recommended 1 ng/µl of DNA, as well as the bioinformatics pipeline should be adjusted for the base errors and higher duplication rate in the DBS data. The quality of the data obtained from the WB samples was as expected based on manufactures recommendations and on previous samples processed at our facility.

When comparing the number of variants detected in the datasets we see a much higher number of total variants called in the DBS samples than in the WB samples, the overlap is 28% between WB variants and DBS variants. This discordancy is reduced after filtering the variants by quality and read depth (Fig. 1), which gives a more uniform number of calls in the individual samples, and increases the overlap to 97% between WB variants and DBS variants (Table 2), and a genotype concordance rate of 96 and 89% for SNPs and indels, respectively. The overlap between annotated WB variants and DBS variants is 94%. Interestingly, we see a slightly higher number of *high* and *moderate* impact variants in the DBS data than in the WB data. We did some additional quality filtering of the variants, which showed that a more stringent filter (read depth >60 and variant call quality >60) removes 37% of the high impact variants in the DBS data and only 16% in the WB data, which indicates a lower overall quality of the DBS data. Therefore, the discordance between genotypes is likely due to false positive calls in the DBS data rather then false negative calls in the WB data. Future studies should include re-genotyping of some of the variants e.g., rs144261584, in order to confirm true variants.

As expected, the success of performing molecular phasing is highly dependant on the molecular lengths of the DNA, which is also shown in our data. The molecular length of the DNA obtained from WB is five times the lengths of the DNA obtained from DBS, and results in higher N50 phase block lengths. However, in average we do see 11% of the DNA from the DBS samples in molecules >20 kb, and between 40 and 58% of the SNPs have been phased, as well as between 21 and 42% of the genes (<100 kb) have been phased. Therefore, using linked-reads to get a better assembly and to get phasing information does seem like a good idea when considering DBS samples, since it adds to the quality of the assembly and in many cases may give the haplotypes accurately.

Linked-reads may be essential for detection of specific diplotypes when compound heterozygosity plays a role in the pathogenesis of disease such as PCD. A previous study of PCD in the Faroe Islands identified four different PCD related variants within *SLC22A5* and a risk-haplotype (RH), where the rs72552725:A > G/RH genotype was the most prevalent (1:600) [13]. Only one of these variants (rs72552725) was called in the trio as shown in supplementary Table 1 (heterozygote in the proband and father, and homozygote with the reference in the mother). Thirteen other variants were called in the trio and all predicted to be benign. No novel pathogenic variant was observed, hence we were not able to identify the second *SLC22A5* variant in the proband, and therefore unable to fully explain the genetic component of the phenotype. Further, as no pathogenic variant within *SLC22A5* was detected in the mother, we are not able to conclude on whether compound heterozygosity may cause the phenotype of the proband. Patients with a single heterozygous pathogenic variant in *SLC22A5* were also identified in Rasmussen et al. 2010, and subsequent analyses of two closely linked microsatellites (RH) showed that these patients shared the same haplotype on the variant negative allele [13]. However, the two microsatellites are not covered in the current DBS and WB data sets, therefore, future studies may include the haplotype, even though, the RH haplotype is also tagged by rs57262206 (NG_008982.1:g.5116G > A) which was homozygote with the reference in the trio.

Some discordance between homozygote and heterozygote calls in the WB and DBS data of the trio is observed; mostly we see homozygotes in the WB data are being called as heterozygotes in the DBS data. Further, all the “no calls” (Supplementary Table 1) are in the DBS data, as well as the large haplotype in the proband’s WB sample is not present in the DBS sample. Again, this may be attributed to the lower coverage or lower quality of the DBS data as in most cases the coverage supports the variants seen in the WB data. Moreover, the lower molecule lengths for DBS (Table 1), may cause an inability to phase the variants, which breaks up the phase blocks and yields smaller haplotypes (Fig. 2).

Using DBS samples as a test bed for molecular phasing needs optimisation of the workflow, as well as the workflow should be tested on larger sample sizes e.g., archive samples with different age. The study shows, as expected, that data obtained from WB samples outperforms the DBS data by e.g., longer molecular lengths and better phasing. Still, we do overcome some of the limitations with DBS samples, such as obtaining enough DNA for NGS analyses, as well as getting long-range information, which adds to the assembly. Concerning the use of DBS for detection of a disease-causing variant in PCD we see that DBS samples perform as well as WB samples in identifying the rs72552725 variant. The discordance of *SLC22A5* genotypes between DBS and WB samples is more likely do to lower quality of the DBS data, as well as the phasing of *SLC22A5* is more complete in the WB data. This also shows that using WB samples together with linked-read sequencing may replace the trio information for haplotype detection. Yet, this conclusion cannot be made for the DBS samples. Further studies have to be made in order to examine how effective phasing with linked-reads is compared to other phasing methods.

## Supplementary information

Supplementary table 1
